# Facial herpes zoster infection precipitated by surgical manipulation of the trigeminal nerve during exploration of the posterior fossa: a case report

**DOI:** 10.4076/1752-1947-3-7813

**Published:** 2009-09-16

**Authors:** Nassir Mansour, Chandrasekaran Kaliaperumal, Kishor A Choudhari

**Affiliations:** 1 Department of Neurosurgery, Regional Neurosciences Unit, Royal Victoria Hospital, Belfast BT12 6BA, UK; 2 National Centre for Neurosurgery, Beaumont Hospital, Dublin-9, Republic of Ireland

## Abstract

**Introduction:**

We present a case of herpes zoster infection (shingles) precipitated by surgical manipulation of the trigeminal nerve root during an attempted microvascular decompression procedure. The pathogenesis of this phenomenon, as well as the importance and role of prophylactic acyclovir in its management, are discussed.

**Case presentation:**

A 54-year-old Caucasian man with a classical long-standing left-sided V2 and V3 division primary trigeminal neuralgia refractory to medical management, underwent posterior fossa exploration for microvascular decompression via a standard retromastoid craniectomy. The patient had immediate and complete relief from pain. Three days after the operation, he developed severely painful vesicles with V2 and V3 dermatomal distribution. Rather than the classical paroxysmal, lancinating type of trigeminal neuralgia, the pain experienced by the patient was of a constant burning nature. A clinical diagnosis of herpes zoster (shingles) was made after smear confirmation from microbiological testing. The patient was commenced on antiviral treatment with acyclovir. His vesicular rash and pain gradually subsided over the next two weeks. He remains asymptomatic one year later.

**Conclusions:**

Postoperative shingles precipitated by trigeminal nerve manipulation during surgery for trigeminal neuralgia can be a distressing and demoralizing experience for the patient. A careful preoperative history, early recognition, and prompt antiviral therapy is necessary.

## Introduction

The herpes zoster virus is known to be associated with trigeminal neuralgia (TN). In some cases it is also implicated in the etiology of TN [1]. A precise pathogenetic mechanism of this association has not been elucidated yet, although shingles that develops after TN can be considered as a type of post-shingles neuropathic pain [1]. We review the possibility of a reactivation of latent varicella zoster infection within the dorsal root ganglion following a microvascular decompression (MVD) procedure resulting in postoperative shingles. The pathogenesis behind postoperative shingles after an MVD operation, as well as the importance and role of prophylactic acyclovir in its management, are discussed in this case report.

## Case presentation

A 54-year-old Caucasian man with a classic long-standing, left-sided maxillary and mandibular (V2 and V3) division primary trigeminal neuralgia, refractory to medical management, underwent a posterior fossa exploration aimed at performing a MVD via a standard retromastoid craniectomy. The trigeminal nerve was identified and exposed from its origin to its entry into the Meckel's cave. Both the sensory and motor nerve rootlets were displayed. No major arterial or venous vascular loop was identified near the dorsal root entry zone (DREZ). The nerve was simply manipulated and pinched using blunt forceps to maximize the procedure's surgical benefit. The patient had immediate and complete pain relief after the operation.

**Figure 1:**
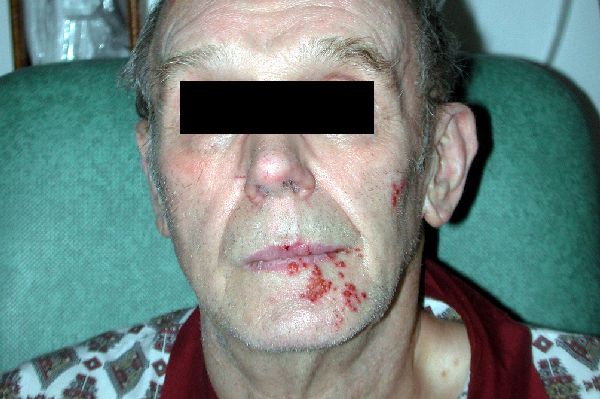
**A photograph of postoperative herpes zoster (shingles) after a microvascular decompression operation**.

Three days after the operation, however, the patient developed severe pain and vesicles along the tracts of V2 and V3 (Figure 1). The pain was characteristically different from the original pain of neuralgia. This pain was of constant burning nature rather than the classical paroxysmal lancinating type of TN, which had completely subsided following surgery. A clinical diagnosis of herpes zoster (shingles) was made, supported by smear reports from microbiological studies. The patient was immunocompetent with no other predisposing factor for developing shingles. He had no history of chickenpox or herpes zoster. The patient was commenced on antiviral treatment with acyclovir. His vesicular rash and pain gradually subsided over the next two weeks. The patient remains asymptomatic one year later.

## Discussion

The most widely accepted theory explaining the aetiopathogenesis of TN is that of vascular compression and arterial pulsations resulting in demyelination at the DREZ. In cases of trigeminal neuralgia without vascular compression or minimal trigeminal nerve root trauma, manipulation is known to provide pain relief [2]. In our experience, the absence of a vascular loop causing microvascular compression on magnetic resonance imaging is not sufficient to exclude patients from MVD, because of the possibility of false negatives [3].

Many patients suffering from TN are also known to have alpha herpesvirus, type-1 herpes simplex virus (HSV-1), or varicella zoster virus (VZV), which are otherwise clinically dormant [4]. HSV-1 has been known to cause periodic reactivation in trigeminal ganglia while the VZV rarely causes its reactivation [3]. The precise mechanism behind the activation of dormant infection by operative manipulation of the trigeminal nerve at the DREZ is difficult to ascertain [4].

Like post-herpetic neuralgia, this phenomenon also appears to occur among elderly individuals. The progressive loss of regulatory control of T lymphocytes associated with the ageing process is thought to play a role in the reactivation of the virus [4].

Herpes zoster (shingles) and varicella (chickenpox) are the two distinctive clinical manifestations of human infection by the VZV [5]. While chickenpox is a primary infection, shingles represents reactivation of a previous infection by VZV. Following the resolution of the primary infection, which usually occurs as childhood chickenpox, the VZV lies dormant in the dorsal root ganglia until a decrease in the cellular immunity triggers its reactivation. This reactivation results in overt herpes zoster infection (shingles) [4]. The virus is known to lie in an inactive form within the Gasserian and geniculate ganglia hence the trigeminal and the facial nerves are the most commonly affected among the cranial nerves.

Post-surgical reactivation of the herpes virus is known to occur following an MVD procedure in patients with post-herpetic neuralgia. Some studies show the incidence of reactivation in patients with postherpetic neuralgia can be as high as 50% [6]. However, the activation from a previously dormant subclinical state of this infection, as in our patient, is a rare occurrence. There was no history or any predisposing factor to explain the occurrence of the herpes virus in our patient. This case therefore suggests that the surgical manipulation could have resulted in the activation of an otherwise asymptomatic herpes infection. This aspect distinguishes our patient's infection from a similar case previously reported by Simms *et al.*[4], and perhaps provides a unique insight into the potential association of a seemingly "non-compressive" TN with latent HZV infection. It also raises the possibility that in patients with "idiopathic" TN, an indolent or subclinical herpes infection could be an aetiological factor.

The observation from autopsy studies that latent herpes infection is common in trigeminal ganglia indirectly supports this view. However, lack of comparative data regarding the incidence of latent herpes infection in patients with or without TN, and relief from the lancinating pain of TN simply by surgical manipulation of the nerve without any antiviral therapy, could argue against this hypothesis. It is not possible to estimate the exact percentage of such cases or to speculate why a surgical intervention causes a clinically occult association to change into clinically overt postoperative shingles in only some patients. Fortunately, this occurrence is extremely rare and runs a benign, self-limiting course, should it happen after uneventful posterior fossa explorations aimed at decompressing the trigeminal nerve from vascular compressions.

Schadelin *et al*. recommend prophylactic acyclovir before MVD procedures in patients with a history of HZV infection [7]. In their study, acyclovir was shown to limit herpes simplex reactivation in a controlled trial to prevent herpes labialis after surgical intervention for trigeminal neuralgia. Out of 14 patients who received acyclovir, unambiguous herpes labialis developed in only one, compared with 12 out of 16 in the placebo group. From our experience and from available information, we acknowledge that in patients with a history of herpes zoster or varicella infection, acyclovir may prevent viral reactivation during the perioperative period. We also express the same recommendation made by Sims *et al*. [4] on the use of prophylactic antiviral treatment for high-risk patients, such as elderly and immunocompromised patients, undergoing MVD, even if there is no history suggestive of such an infection.

## Conclusions

This case report demonstrates that surgical manipulation of the trigeminal nerve during posterior fossa explorations for an MVD procedure has the potential to activate a previously dormant herpes infection. Postoperative shingles precipitated by surgery for TN can be a distressing and demoralizing experience for the patient. Relief from one type of pain just to be replaced by another severe pain can put a dent in the patient's confidence in the surgical intervention, while also prolonging the patient's hospitalization. A careful preoperative history, early recognition and prompt antiviral therapy, as well as making it a point to reassure the patient, are necessary.

## Abbreviations

DREZ: dorsal root entry zone; HSV: herpes simplex virus; MVD: microvascular decompression; TN: trigeminal neuralgia; VZV: varicella zoster virus.

## Consent

Written informed consent was obtained from the patient for publication of this case report and any accompanying images. A copy of the written consent is available for review by the Editor-in-Chief of this journal.

## Competing interests

The authors declare that they have no competing interests.

## Authors' contributions

NM collected the necessary data on the patient and drafted the manuscript. CK prepared the discussion for the case report. KAC is the senior neurosurgeon involved in the overall management of the patient. He also analyzed and revised the manuscript. All authors read and approved final manuscript.
